# Thick Does the Trick: Genesis of Ferroelectricity in 2D GeTe‐Rich (GeTe)_*m*_(Sb_2_Te_3_)_*n*_ Lamellae

**DOI:** 10.1002/advs.202304785

**Published:** 2023-11-21

**Authors:** Stefano Cecchi, Jamo Momand, Daniele Dragoni, Omar Abou El Kheir, Federico Fagiani, Dominik Kriegner, Christian Rinaldi, Fabrizio Arciprete, Vaclav Holý, Bart J. Kooi, Marco Bernasconi, Raffaella Calarco

**Affiliations:** ^1^ Department of Materials Science University of Milano‐Bicocca via R. Cozzi 55 20125 Milano Italy; ^2^ Paul‐Drude‐Institut für Festkörperelektronik Leibniz‐Institut im Forschungsverbund Berlin e.V. Hausvogteiplatz 5‐7 10117 Berlin Germany; ^3^ Zernike Institute for Advanced Materials University of Groningen Nijenborgh 4 9747 AG Groningen The Netherlands; ^4^ Dipartimento di Fisica Politecnico di Milano P.zza Leonardo da Vinci 32 20133 Milano Italy; ^5^ Institute of Solid State and Materials Physics Technische Universität Dresden Helmholtzstr. 10 01069 Dresden Germany; ^6^ Institute of Physics Czech Academy of Sciences Cukrovarnická 10/112 16200 Praha 6 Czech Republic; ^7^ Dipartimento di Fisica Università di Roma “Tor Vergata” Via della Ricerca Scientifica 1 00133 Rome Italy; ^8^ Department of Condensed Matter Physics Faculty of Mathematics and Physics Charles University, Ke Karlovu 5 12116 Praha Czech Republic; ^9^ Institute of Condensed Matter Physics Faculty of Science Masaryk University Kotlářská 2 611 37 Brno Czech Republic; ^10^ CNR Institute for Microelectronics and Microsystems–IMM Consiglio Nazionale delle Ricerche Via del Fosso del Cavaliere 100 00133 Roma Italy

**Keywords:** 2D ferroelectrics, van der Waals, molecular beam epitaxy, phase‐change materials, density functional theory calculations

## Abstract

The possibility to engineer (GeTe)_*m*_(Sb_2_Te_3_)_*n*_ phase‐change materials to co‐host ferroelectricity is extremely attractive. The combination of these functionalities holds great technological impact, potentially enabling the design of novel multifunctional devices. Here an experimental and theoretical study of epitaxial (GeTe)_*m*_(Sb_2_Te_3_)_*n*_ with GeTe‐rich composition is presented. These layered films feature a tunable distribution of (GeTe)_*m*_(Sb_2_Te_3_)_1_ blocks of different sizes. Breakthrough evidence of ferroelectric displacement in thick (GeTe)_*m*_(Sb_2_Te_3_)_1_ lamellae is provided. The density functional theory calculations suggest the formation of a tilted (GeTe)_*m*_ slab sandwiched in GeTe‐rich blocks. That is, the net ferroelectric polarization is confined almost in‐plane, representing an unprecedented case between 2D and bulk ferroelectric materials. The ferroelectric behavior is confirmed by piezoresponse force microscopy and electroresistive measurements. The resilience of the quasi van der Waals character of the films, regardless of their composition, is also demonstrated. Hence, the material developed hereby gathers in a unique 2D platform the phase‐change and ferroelectric switching properties, paving the way for the conception of innovative device architectures.

## Graphical abstract

The genesis of ferroelectricity in thick (GeTe)_*m*_(Sb_2_Te_3_)_1_ lamellae is uncovered. The resilience of the quasi 2D character of GeTe‐rich (GeTe)_*m*_(Sb_2_Te_3_)_*n*_ films is demonstrated. It paves the way for the design of novel van der Waals heterostructures and multifunctional devices.

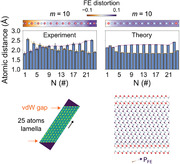

## 1 Introduction

The advent of 2D materials, ignited by the isolation of graphene,^[^ ^1^, ^2^ ^]^ redefined the material science of the last decade. The multitude of newly discovered layered systems and their heterostructures hold out tremendous expectations in diverse technological fields.^[^ ^3^, ^4^, ^5^, ^6^, ^7^, ^8^, ^9^ ^]^ Among the portfolio of synthesis techniques suitable for 2D materials, van der Waals (vdW) epitaxy^[^ ^10^ ^]^ ensures high‐quality, purity, and scalability, all central in the pathway toward integration with microelectronic technology.

In recent years, molecular beam epitaxy (MBE) has been used to exploit vdW epitaxy for a variety of layered compounds and heterostructures.^[^ ^11^ ^]^ In particular, great effort has been devoted to the MBE growth of layered materials in the Ge‐Sb‐Te chalcogenide family, which gathers a rich multifunctional playground of topological,^[^ ^12^ ^]^ thermoelectric,^[^ ^13^, ^14^ ^]^ and phase‐change properties.^[^ ^15^, ^16^ ^]^ The pseudo‐binary compound Ge_2_Sb_2_Te_5_, for instance, is utilized in phase‐change memory devices, that nowadays are a consolidated non‐volatile technology.^[^ ^17^, ^18^, ^19^ ^]^ Recently, (GeTe)_*m*_(Sb_2_Te_3_)_*n*_ (GST) alloys have fueled interest as the most prominent candidates for storage‐class memories,^[^ ^20^ ^]^ brain‐inspired and in‐memory computing.^[^ ^21^, ^22^, ^23^ ^]^ The parent binary compound GeTe is also a well known ferroelectric (FE) semiconductor^[^ ^24^, ^25^ ^]^ and possesses giant Rashba spin splitting of its bulk bands,^[^ ^26^, ^27^ ^]^ although it is not properly a layered material.

In this work we show that epitaxial (epi‐) GST with GeTe‐rich composition fabricated by MBE exhibits a FE behavior, while still retaining a layered geometry. This outcome originates from a comprehensive experimental and theoretical analysis of the structure of GST lamellae as a function of composition, which provides breakthrough evidence of the emergence of FE displacement in very thick GST lamellae. The FE behavior of epi‐GST is confirmed by piezoresponse force microscopy (PFM) and electroresistive measurements. Moreover, we demonstrate the robustness of the layered structure peculiar to highly ordered GST alloys, which allows for the vdW epitaxy of lamellae up to unexpectedly large thickness. Thus, our work brings to light the additional FE functionality enclosed in GeTe‐rich epi‐GST which, in combination with its resilient 2D character, opens the way for the design of novel multifunctional devices.

## 2 Local Structure and Ferroelectricity in (GeTe)_*m*_(Sb_2_Te_3_)_1_ Lamellae

The task anticipated in the introduction is ambitious in the case of GST, being these alloys highly disordered systems intrinsically affected by a large amount of defects.^[^ ^28^, ^29^ ^]^ Therefore, in the following we benefit from the very high crystal and structural quality accomplished in layered epi‐GST fabricated by MBE.^[^ ^30^, ^31^ ^]^ Then, we were able to probe by high‐angle annular dark‐field scanning transmission electron microscopy (HAADF‐STEM) the atomic structure of GST lamellae of various sizes hosted in a GeTe‐rich film, gaining access to their intralamellar FE distortion. Indeed, while MBE guarantees to attain crystalline GST with trigonal or cubic structure and vacancies ordered into layers,^[^ ^32^ ^]^ the epitaxial films still feature compositional disorder, that is, a distribution of GST blocks (i.e., lamellae) with a different number of atomic layers is present.^[^ ^30^, ^33^ ^]^

Details about the MBE growth of the epi‐GST films are reported in Section 6. Starting from the optimized parameters for Ge_2_Sb_2_Te_5_ (sample GST225),^[^ ^31^ ^]^ the GST composition was controlled by increasing the atomic flux of Ge while keeping fixed the other growth parameters (sample GST528), thus providing an excess of Ge (see **Table** **1**). The growth experiments were observed in situ by reflection high‐energy electron diffraction (RHEED). An extensive ex situ structural characterization of the epi‐GST films is presented in the second part of this report, offering a thorough picture of the structural characteristics of epi‐GST layered alloys with increased GeTe content (Section 3), and of their resilient 2D character (Section 4).

**Table 1:** Relevant growth and structural parameters extracted from the XRD radial scans and RHEED movies for the epi‐GST samples. $\Phi_{\text{Ge}}^{\text{norm}}$ is the normalized Ge flux employed in the growth experiments (that of GST225 is taken as a reference). Φ_Ge_/Φ_Te_ is the Ge/Te flux ratio. *m* and *D*_GST_ are the estimated number of GeTe bilayers (BLs) in the average GST block and its thickness (including the Te–Te gap between lamellae), respectively. *D*_GST_ is calculated from the separation between the 2nd order main peak and VL peak. Statistical errors for the Q_*z*_ position of the GST main and VL peaks are below 0.002 Å^−1^. $d_{\text{RHEED}}^{\text{relax}}$ and *L*_decay_ are the fitted GST lattice plane spacing after relaxation and the decay length of the exponential fit. The statistical errors for $d_{\text{RHEED}}^{\text{relax}}$ and *L*_decay_ are below 0.001 Å and 0.2 nm, respectively.

	$\Phi_{\text{Ge}}^{\text{norm}}$	Φ_Ge_/Φ_Te_	*m*	*D*_GST_	$d_{\text{RHEED}}^{\text{relax}}$	*L*_decay_
			(#)	[Å]	[Å]	[nm]
GST225	1	0.28	1.97	17.62	2.455	1.8
GST528	2.94	0.82	4.90	28.27	2.441	2.7

**Figure 1:**
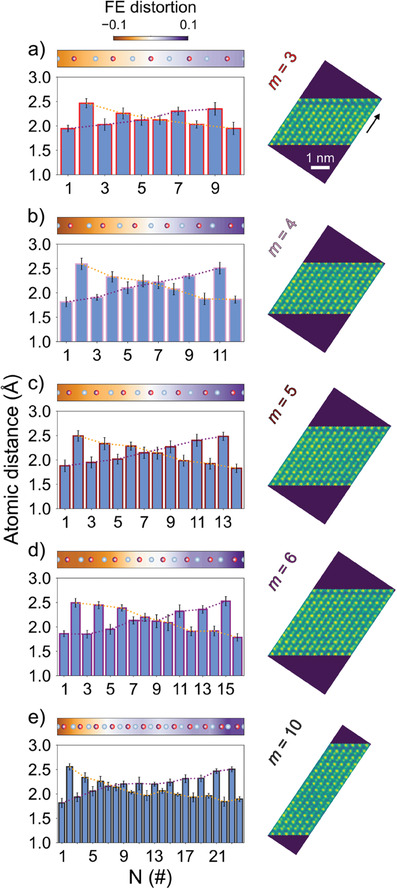
Experimental measurements of the local structure and ferroelectric distortion in (GeTe)_*m*_(Sb_2_Te_3_)_1_ lamellae. Atomic plane distances (light blue bars) of GST blocks with a) *m* = 3, b) *m* = 4, c) *m* = 5, d) *m* = 6, and e) *m* = 10, extracted from high resolution STEM micrographs of sample GST528. Te and Ge/Sb atoms are sketched in gray and violet/red, respectively. The portion of lamella used for the analysis is also shown. The black arrow in (a) indicates the direction along with the profile is calculated. The colormap represents the FE distortion along the slabs. Dotted orange and purple lines highlight the presence of an FE domain wall, which for the thin slabs with inversion symmetry lies at the center of the slab. For the block with *m* = 10 the domain wall is displaced from the center.

A precise measurement of the atomic plane distances in GST blocks with *m* = 3–6 and *n* = 1 was extracted from high resolution STEM micrographs of sample GST528 (*m* and *n* being the numbers of GeTe and Sb_2_Te_3_ units). The method is explained in Section 6. The *N* = (2*m* + 4) distances in each slab are plotted in **Figure** **1**a–d. These have been measured along the direction indicated by the black arrow in Figure 1a. The dotted orange and purple guide lines facilitate to visualize the complex structure of GST lamellae. The local FE distortion is defined as the relative displacement of each Ge/Sb plane with respect to the central position between its neighbor Te planes (see Equation (S1), Supporting Information). The profile of the atomic plane distances is undoubtedly symmetric (X‐shaped profile). Shorter and longer distances alternate, forming BLs with decreasing FE distortion from the edges of the lamellas toward the center (see the colormap in the panels), where de facto a FE domain wall is engraved. Furthermore, such distortion shows dependence upon composition of the lamellae.

**Figure 2:**
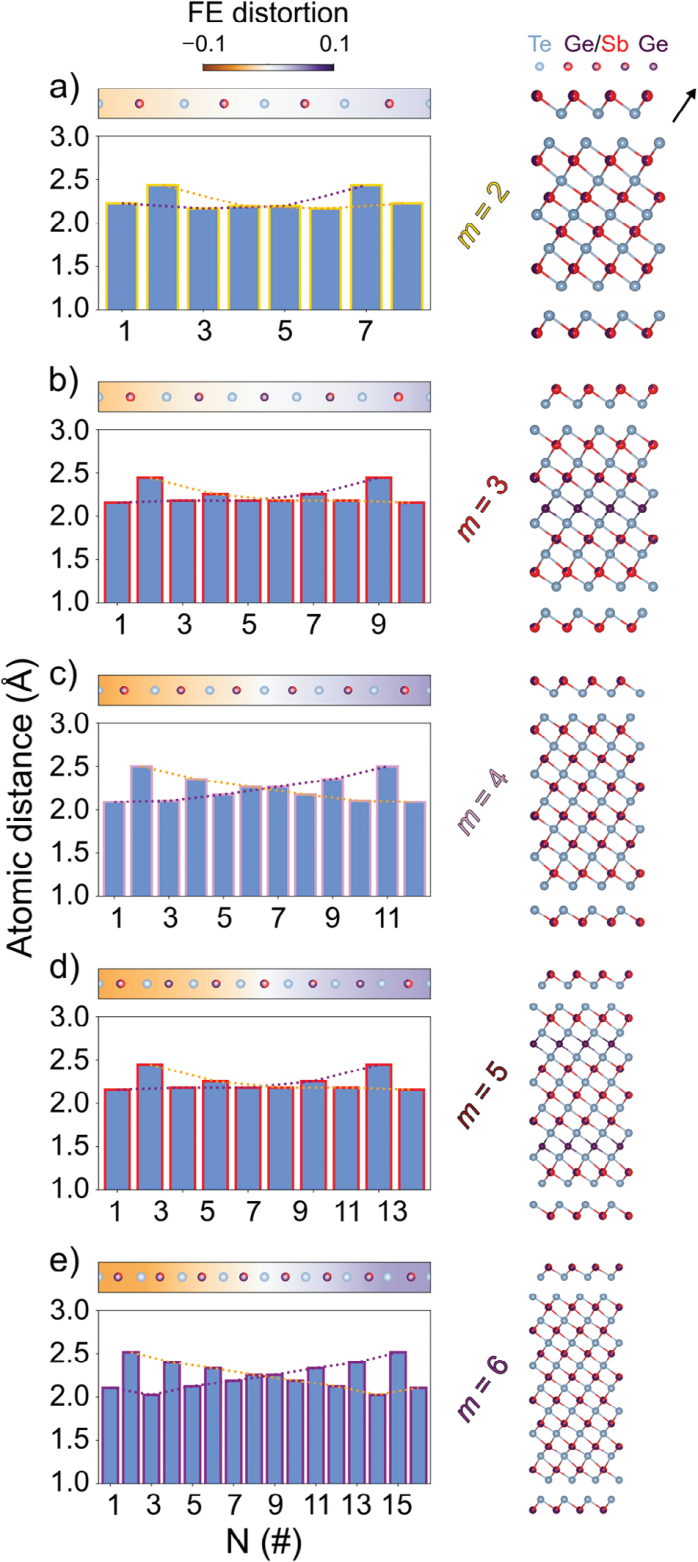
Calculations of the local structure and ferroelectric distortion in (GeTe)_*m*_(Sb_2_Te_3_)_1_ lamellae. Atomic plane distances (light blue bars) of the pure (GeTe)_*m*_(Sb_2_Te_3_)_1_ pseudobinary systems with: a) *m* = 2, b) *m* = 3, c) *m* = 4, d) *m* = 5, and e) *m* = 6, calculated in the framework of DFT. Te, Ge, and Ge/Sb atoms are sketched in gray, violet, and violet/red, respectively. The models include disorder in the Ge/Sb sublattices. The schematics of the structures after relaxation are also shown, in which the Ge/Sb composition in each cationic plane of the layered crystals is displayed by the proportion of violet/red colors. The black arrow in (a) indicates the direction along with the profile is calculated. The colormap represents the FE distortion along the slabs. Dotted orange and purple lines highlight the presence of an FE domain wall, which for the thin slabs with inversion symmetry lies at the center of the slab.

Aiming at unraveling the structural properties of highly ordered GeTe‐rich compounds, while supporting the picture inferred by STEM analysis, we employed density functional theory (DFT). Calculations of the (GeTe)_*m*_(Sb_2_Te_3_)_1_ pseudobinary systems with *m* ranging between 2 and 6 have been performed. Details are given in the Experimental Section and the Supporting Information. The theoretical structural data in **Table** **2**, and Tables S1 and S2, Supporting Information, were obtained by modeling crystalline Ge_2_Sb_2_Te_5_, Ge_3_Sb_2_Te_6_, Ge_4_Sb_2_Te_7_, Ge_5_Sb_2_Te_8_, and Ge_6_Sb_2_Te_9_ with 2 × 2 × 1 supercells (in the hexagonal setting) including disorder in the Ge/Sb sublattices. The schematics of the structures after relaxation, in which the Ge/Sb composition in each cationic plane of the layered crystals is indicated, are shown in the right panels of **Figure** **2**. The rationale behind the choice of the modeled slabs is described in Section 6. The theoretical equilibrium lattice parameters of the different compounds are compared with experimental data from refs. [34, 35, 36] in Table S1, Supporting Information.

**Table 2:** Atomic plane distances of the average (GeTe)_*m*_(Sb_2_Te_3_)_1_ block fitted for GST225 and GST528 samples. Equivalent distance parameters obtained from DFT calculations and averaged over the block distribution from STEM are also reported (DFT_STEM_). The *d*_vdW_, *d*_1_, and *d*_2_ parameters are the Te–Te distances at the gap between neighboring blocks, in the block close to the gap and in the (GeTe)_*m*_ slab at the center of the block, respectively. The *d*_*z*1_ and *d*_*z*2_ parameters are the distances between Te and Ge/Sb layers at the edges of the GST block and in the (GeTe)_*m*_ slab. Statistical errors for the fitted distances, obtained from the least square optimization, are below 1% of the respective value.

	GST225		GST528	
Distances	XRD fit	DFT_STEM_	XRD fit	DFT_STEM_
	[Å]	[Å]	[Å]	[Å]
*d*_vdW_	2.98	2.92	3.00	2.95
*d*_1_	3.66	3.66	3.62	3.65
*d*_2_	3.59	3.43	3.59	3.45
*d*_*z*1_	1.49	1.65	1.33	1.61
*d*_*z*2_	1.54	1.66	1.47	1.64

To compare with experiments, we extracted from the theoretical models information equivalent to the STEM profiles (see left panels of Figure 2). The resulting equilibrium structures feature a reflection symmetry with respect to the central layer of the slab, which is either a cationic (Ge/Sb) or anionic (Te) layer. The upper and lower half slabs spotlight the formation of BLs with shorter and longer Te–Ge/Sb distances as occurs in trigonal GeTe. The orientation of the FE polarization is opposite in the two half slabs. Such distortion with respect to the cubic octahedral geometry is mostly enhanced close to the vdW gap and increases in magnitude with the content of GeTe in the (GeTe)_*m*_(Sb_2_Te_3_)_1_ pseudobinary alloy, although this trend is not always monotonous in the series with *m* = 2–6. The agreement between our measurements and the atomic configurations foreseen by DFT is remarkably good.

As already stated, albeit the increase of the Peierls distortion with the thickness of the GST block is described by DFT, the net FE polarization is zero because of the reflection symmetry at the center of the lamellae. On the contrary, here we prove for the first time that in thick GST lamellae such symmetry is broken, leading to the genesis of a sizable FE polarization. This finding resulted from the STEM analysis on a 25‐atomic layer thick GST lamella (*m* = 10), shown in panel (e) of Figure 1. Another example with *m* = 13 is given in the Supporting Information (Figure S1, Supporting Information). In these cases the profile still possesses a FE domain wall, which is, however, not at the center of the slab. Consequently, the two halves of the block feature different local FE polarization. Such atomic configuration, which is definitely more complex compared to the structure of trigonal GeTe, lead to a net FE polarization. The magnitude of the FE displacement in the lamella with *m* = 10, as compared with that of an equivalent number of GeTe BLs measured in an epitaxial sample, is about a third (see Table S4, Supporting Information). Nevertheless, we are dealing with a system that potentially bridges the phase‐change and FE switching properties in a unique 2D platform (see Section 4).

**Figure 3:**
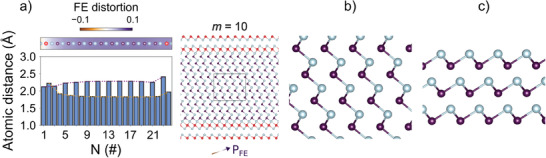
Calculations of the local structure and ferroelectric distortion in a (GeTe)_10_(Sb_2_Te_3_)_1_ lamella. a) Atomic plane distances (light blue bars) of a thick GST lamella with *m* = 10, calculated in the framework of DFT. Te, Ge, and Sb atoms are sketched in gray, violet, and red, respectively. The structure has no disorder in the cationic sublattice. The fully relaxed configuration is also shown, consisting of a tilted (GeTe)_*m*_ slab sandwiched in the lamella by SbTe capping layers on both sides. The shortest Ge–Te bonds in the lamella are visualized by sticks. The direction of the FE polarization is indicated by a graded color arrow. The colormap represents the FE distortion along the slab. Dotted orange and purple lines highlight the presence of an FE domain wall, which in this case is very close to one edge of the lamella. b) Higher magnification sketch of the central region of the Ge_10_Sb_2_Te_13_ lamella. c) Schematic of bulk GeTe computed by DFT.

The formation of a net FE polarization in GeTe‐rich GST lamellae is confirmed by DFT calculations for a thick lamella composed by 25‐atomic layers, corresponding to a composition of Ge_10_Sb_2_Te_13_. For computational reasons, we considered a centrosymmetric Kooi‐like configuration with no intermixing in the cationic sublattice. This structure turns out to be mechanically unstable and spontaneously relaxes to a new configuration which breaks inversion symmetry (see right panel of **Figure** **3**a). The energy of this structure is 6 meV/at lower than the centrosymmetric starting configuration. Details are provided in the Experimental Section.

Again, we extracted the equivalent atomic plane distance profile from the theoretical model (see left panel of Figure 3a). The sequence of short‐long distances is strongly asymmetric with respect to the center of the lamella, therefore a net FE displacement appears. As opposed to experiments, the short–long distance pairs are all very similar through the lamella, while it still accommodates a domain wall very close to the edge of the lamella. This discrepancy might arise from the lack of disorder in the cationic sublattice in the theoretical model.

Nevertheless, the DFT calculations shed light on a surprising feature of these FE lamellae. The structure recalls a pure (GeTe)_*m*_ slab encapsulated in SbTe capping layers on both sides. Unexpectedly, the orientation of the GeTe BLs is tilted with respect to the *c*‐axis of the lamella, as shown in Figure 3a,b. For the sake of comparison, the structure of a bulk GeTe is depicted in Figure 3c (details are reported in Table S3, Supporting Information). That is, the FE polarization, which is normal to the BL structure, is close to the in‐plane direction (≈71° tilt with respect to the direction normal to the lamella). The confinement of the FE displacement found here is displayed by other FE materials in their 2D phases,^[^ ^37^, ^38^ ^]^ and has been predicted theoretically but so far not observed experimentally for 2D GeTe.^[^ ^39^ ^]^ Thus, we are convinced to have discovered a material structure lying at the transition between 2D and 3D FE materials.

**Figure 4:**
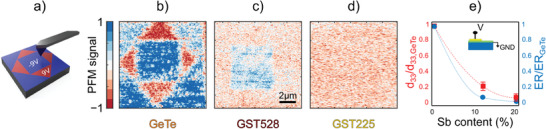
Investigation of ferroelectricity of the epi‐GST films grown on Si(111). a) The surface of each sample was poled with the conductive tip of the AFM at ±9 V, in a diamond‐like fashion over three squares with a side of 10, 7, and 5 μm. b–d) Out‐of‐plane PFM signal measured on the surface of GeTe, GST528, and GST225 samples normalized to the signal of pure GeTe. Thus, the values −1 and +1 correspond to the maximum outward (red) and inward (blue) FE polarization detected on pure GeTe. e) Red squares show the PFM contrast measured in panels (b–d) between outward and inward polarization versus the concentration of Sb. Blue dots report the electroresistance (ER) measured on the same samples through Ti contacts deposited on the film surface (inset), normalized to the signal of pure GeTe. Dashed lines serve as guides for the eye.

To validate our analysis, the measurement of the FE properties of epi‐GST is crucial. The piezoelectric behavior versus film composition was investigated by PFM measurements, following the approach used to demonstrate ferroelectricity in GeTe epitaxial films.^[^ ^27^ ^]^ Ferroelectric domains were patterned on the surface of each sample according to the geometry reported in **Figure** **4**a. The conductive tip of the atomic force microscope (AFM) was biased at −9 V (+9 V) to set the polarization in the outward (inward) direction (see Section 6).

Figure 4b–d reports the out‐of‐plane piezomechanical response of a reference epi‐GeTe, GST528, and GST225 samples as the result of such domain patterning. The signal is here normalized to the one of pure GeTe at saturation, in order to highlight the material‐dependent response. The progressive decrease of the PFM contrast between different domains in the out‐of‐plane direction is summarized in Figure 4e as the normalized piezoelectric coefficient *d*_33_ as a function of the percentage of Sb in GST. Although relatively weak, the sample GST528 still presents a ferroelectric response in the out‐of‐plane direction (Figure 4c), while effectively no contrast is observed between outward and inward regions for GST225 (Figure 4d). The FE behavior of the films was also tested locally by measuring ferroelectric hysteresis loops (an example for sample GST528 is shown in Figure S2, Supporting Information).

Further confirmation of this trend is provided by electroresistive measurements on the same samples after the deposition of metallic contacts on top (see the Experimental Section). As previously demonstrated in ref. [27], the resistance of a metal/ferroelectric‐semiconductor heterojunction depends on the orientation of the component of the polarization perpendicular to the interface due to the role of the polarization charge. Thus, the FE switching caused by voltage pulses applied through the junction (inset of Figure 4e) manifests as an electroresistance, defined as the difference between the higher and the lower saturation values over the lower value. Noteworthy, Figure 4e reports a monotonic decrease of the electroresistance as a function of the content of Sb.

## 3 GeTe‐Rich Epi‐GST Layered Alloys: Structural Properties

The set of data presented in the previous section yields an in‐depth understanding of the structure of single (GeTe)_*m*_(Sb_2_Te_3_)_1_ lamellae. Here below, we complement this analysis with an overview of the structural characteristics of epi‐GST layered alloys with increased GeTe content at a layer scale.

**Figure 5:**
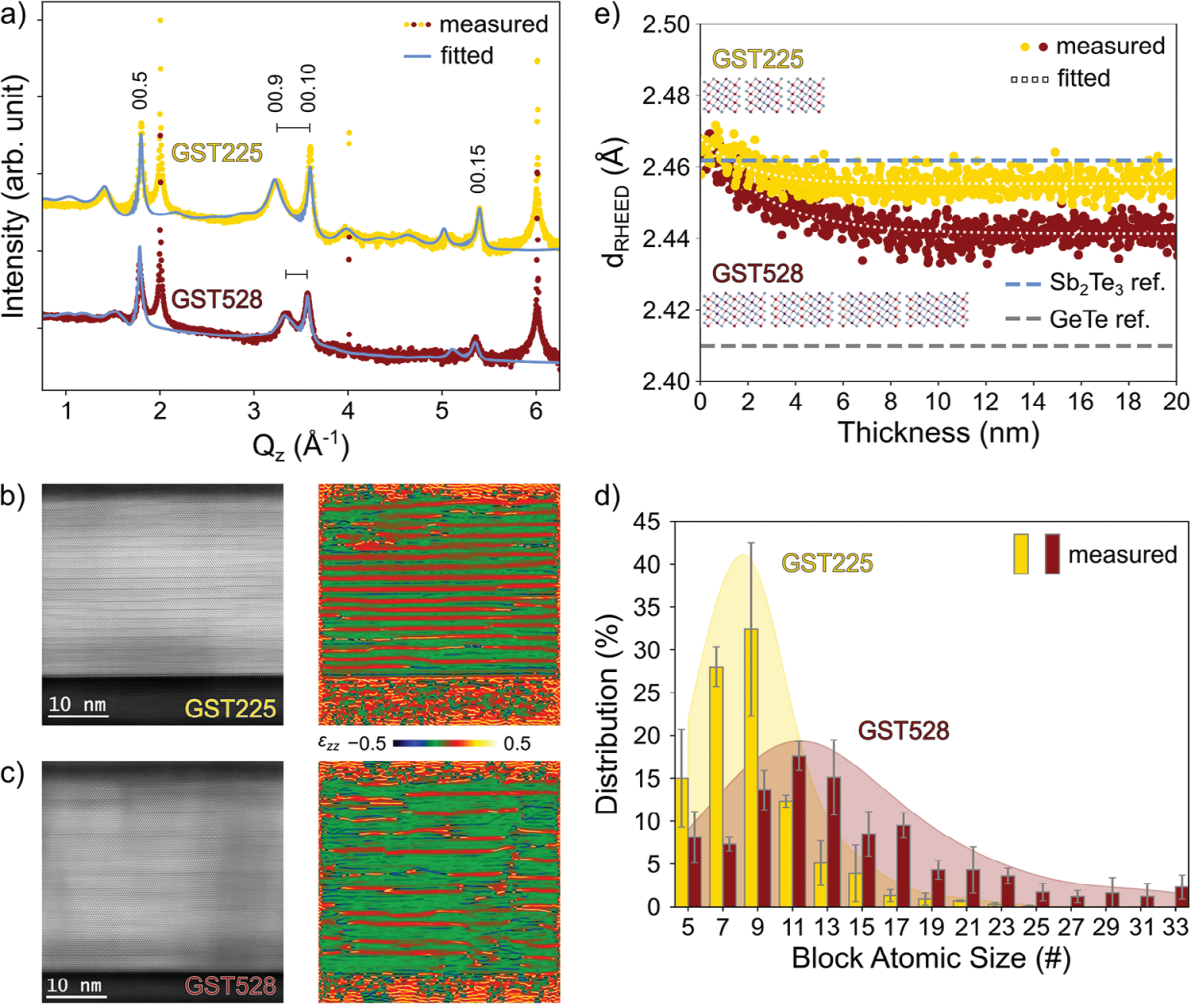
Structural characterization of the epi‐GST films grown on Si(111). Reference GST225 curves are plotted in yellow, while dark red curves correspond to GST528. a) Radial scans, plotted in logarithmic scale and vertically shifted for clarity. The sharp peaks at ≈2, 4, and 6 Å^−1^ correspond to the 111, 222, and 333 Bragg reflections of the Si substrate. The more intense Bragg reflections of GST225 are labeled. The alloy compositions is estimated from the average GST block size measured by the Q_*z*_ separation between the GST main and VL peaks (black bars). The fitted curves (solid light blue lines) are superimposed to the measurements. b,c) Exemplary HAADF‐STEM micrographs used for the analysis (left) and corresponding GPA images (right). The color contrast in GPA plots is given by the different relative displacement ϵ_*zz*_ between shorter(longer) Ge/Sb‐Te(Te‐Te) interplanar distances. d) Distribution of (GeTe)_*m*_(Sb_2_Te_3_)_1_ block atomic size in GST225 (yellow bars) and GST528 (dark red bars) samples. The KDE of the distributions is also plotted as guide for the eye. e) Lattice plane spacing *d*_RHEED_ of the growing GST225 (yellow) and GST528 (dark red) films during deposition of the first 20 nm. The fitted curves (dotted white) are superimposed to the measurements. The bulk lattice plane spacing of Sb_2_Te_3_^[^ ^41^ ^]^ and GeTe^[^ ^42^ ^]^ are plotted as reference (light blue and gray dashed lines, respectively). Ge_2_Sb_2_Te_5_ and Ge_5_Sb_2_Te_8_ block structures, rescaled with the corresponding average block thickness D_GST_ in Table 1, are shown to highlight the relationship between the number of lamellae and the relaxation of the film.

The X‐ray diffraction (XRD) radial scans of GST225 and GST528 films are compared in panel (a) of **Figure** **5**. The sharp peaks at ≈2, 4, and 6 Å^−1^ correspond to the 111, 222, and 333 Bragg reflections of the Si(111) substrate. With respect to GST225 (yellow curve), the effect of the increased Ge flux is here underlined. Remarkably, both curves show the typical pattern of epi‐GST: the more intense and sharper crystal main peaks are accompanied by broader vacancy layer (VL) peaks, resulting from the disordered, still layered, structure of GST.^[^ ^30^, ^33^ ^]^ While the features attributed to GST are qualitatively similar, the *Q*_*z*_ separation between the GST main and VL peaks decreases (see black bars in Figure 5a), testifying to the larger atomic thickness of the average GST block, that we indicate as *D*_GST_ hereafter (reported in Table 1). The corresponding average composition indicated in the figure was estimated using the approach suggested by Da Silva and coworkers for the homologous series (GeTe)_*m*_(Sb_2_Te_3_)_*n*_.^[^ ^40^ ^]^ More details are found in the Experimental Section. The sample with more GeTe (GST528) has an average composition close to Ge_5_Sb_2_Te_8_ (dark red curve).

As shown in Figure 1, HAADF‐STEM measurements were performed on the samples. While we first focused on the structure of GST lamellae of different sizes found in sample GST528, the goal of the analysis hereby is to inspect the structure of the two films, validating the average block size estimation by XRD. Similar to a previous work by Momand et al. on Sb_2_Te_3_/GeTe superlattice structures,^[^ ^43^ ^]^ Figure 5d shows the block atomic‐plane distribution in the two GST films (yellow and dark red bars for GST225 and GST528, respectively) as extrapolated by applying geometric phase analysis (GPA) to several micrographs.^[^ ^44^, ^45^ ^]^ The kernel density estimate (KDE) of the distributions is plotted in the background as guide for the eye. The same was repeated for GST225 on a large scale image (Figure S3, Supporting Information), which consolidates our methodology. Examples of HAADF‐STEM micrographs used for the analysis (left), and the corresponding GPA images (right), are shown in Figure 5b for GST225 and Figure 5c for GST528, respectively. The red regions in the GPA images correspond to the vacancy layer patterns as derived from the analysis.

The difference between the layered structure of the two films and the corresponding block distributions is striking. For GST225, the distribution is centered at a block size of 9‐atomic layers, which also corresponds to the average value in the film and matches the *D*_GST_ calculated from the XRD radial scan (Table 1). Its dispersion is relatively limited, with more than 95% of the blocks having a thickness below 17‐atomic layers. Similar investigations reported by Mio et al.^[^ ^33^ ^]^ led to qualitatively comparable block distributions. On the contrary, the reduced number of vacancy layers per unit length in the GeTe‐rich sample (see Figure 5c) determines a much broader distribution of the slab thicknesses, with the presence of very thick lamellae stabilized by epitaxy. In fact, GST blocks as thick as 33‐atomic layers (which translates to ≈6 nm) are stacked in the film. It is worth to stress that, to the best of the authors knowledge, this is the first report of as‐grown ordered GST exhibiting such characteristics. The broader block distribution displays a slightly wavy envelope, still featuring a maximum at 11‐atomic layers. Nevertheless the film's average block size, falling between 13‐ and 15‐atomic layers, is in fairly good agreement with the estimation by XRD. The modest discrepancy between XRD and GPA for GST528 sample can be ascribed to the limited statistics accessible for films with larger average block size. The different FE behavior of the two samples, and in particular the absence of a piezomechanical response for sample GST225, are intimately linked to their block atomic‐plane distributions.

In close analogy to the statistical modeling developed to simulate the XRD radial scans of V_2 +*x*_(Te,Se)_3_ epitaxial films,^[^ ^14^, ^46^, ^47^ ^]^ a fit routine for layered GST was devised. Here, we aim to extract complementary information on the structure of GST lamellae in the films. Yet, severe differences exist between the structure of layered binary tellurides and epi‐GST, drastically hampering the opportunity for accurate simulations.^[^ ^48^ ^]^ The intrinsic disorder owned by the layered structure of GST gives rise to its largely featureless XRD pattern. As a consequence, similar characteristic features are present in the radial scans of epi‐GST films almost independently of the alloy composition (Figure 5a), which complicates the modeling. To date, leveraging on the reduced compositional disorder achieved for epi‐GST on vicinal Si(111) surfaces,^[^ ^49^ ^]^ a Monte Carlo method was implemented for alloy compositions close to Ge_1_Sb_2_Te_4_.

The model assumes random sequences of (GeTe)_*m*_(Sb_2_Te_3_)_1_ blocks forming the crystal. Based on DFT calculations, mirror‐symmetric GST structures described by five atomic distance parameters were conceived (see Figure S4, Supporting Information). The random numbers *m* of GeTe BLs in consecutive GST blocks are statistically independent. The size of the GST blocks in the sequences is described in the model by a distribution function, which is given as an entry parameter. We recall that in previous applications of the present modeling the random variables were described by Gamma distributions, introducing additional fitting parameters to the calculations. In this work, instead, the block atomic‐plane distributions measured by STEM for GST225 and GST528 films (Figure 5d) were used as input in the simulations, simplifying considerably the fitting procedure. Details about the modeling are reported in the Supporting Information.

Applying this constraint, we successfully reproduced the main features in the radial scans of the two GST samples. The fitted curves are plotted in Figure 5a (solid light blue lines) together with the experimental ones. Noteworthy, the average atomic distances of the (GeTe)_*m*_(Sb_2_Te_3_)_1_ blocks in GST225 and GST528 films were fitted, granting unprecedented insights on the structure of these alloys which could hardly be retrieved on a large scale, for example from STEM micrographs. The calculated distances are reported in Table 2 and compared to equivalent parameters adapted from DFT calculations (see Experimental Section). The Te–Te distance at the gap between neighboring blocks (*d*_vdW_) is smaller in the GST225 sample. Correspondingly, the distance *d*_1_ between the Te layers in the GST block close to the gap shows an opposite behavior, suggesting a correlation with *d*_vdW_. At first glance it may imply an overall larger depletion of the vacancy layers in GST225.^[^ ^30^, ^50^ ^]^ Outstandingly, DFT calculations unfold a more general relationship between the composition and geometry of the pseudobinary GST blocks, which is in qualitative agreement with XRD simulations (see Table 2; Table S2, Supporting Information). The Te–Te distance in the (GeTe)_*m*_ slab at the center of the block (*d*_2_) is instead unchanged between the two samples, while in both cases *d*_2_ is smaller than *d*_1_. The same relation between *d*_1_ and *d*_2_ applies to all DFT models. Finally, concerning the Te–Ge/Sb distances close to the gap (*d*_*z*1_) and in the (GeTe)_*m*_ slab (*d*_*z*2_), we notice that *d*_*z*1_ is smaller than *d*_*z*2_, while both distances decrease with increasing GeTe content. Even though the fitted values are smaller compared to the corresponding DFT ones, a similar trend is found. Also, the tiny change of *d*_*z*1_/*d*_1_ and *d*_*z*2_/*d*_2_ ratios, decreasing from GST225 to GST528, drags us to consistently speculate that in GeTe‐rich GST alloys the (GeTe)_*m*_ slab in the lamellae may tend to mimic the structure of bulk GeTe. Besides, the modeling provides an independent proof of the block size(composition)‐dependent change of the geometry of GST lamellae, disclosing their arising Peierls distortion as more GeTe BLs are packed in the slab.

## 4 Strain Relaxation

This final section is devoted to elucidate the relaxation behavior of the GST films on Sb‐passivated Si(111), which brings to light the persistent quasi vdW nature of highly ordered GST alloys. In layered materials, the term “quasi vdW” describes a stronger interaction between adjacent lamellae, which is not purely a vdW character but features a contribution from axial bonds. This can also be associated with the so‐called metavalent bonding.^[^ ^51^ ^]^ The low lattice mismatch case of epi‐GST on InAs(111) has been very recently studied.^[^ ^52^ ^]^ The $\left\{11\bar{2}\right\}$ lattice plane spacing of the growing GST films (*d*_RHEED_) is shown in Figure 5e. The data for GST225 and GST528 samples are plotted in yellow and dark red, respectively, along with fitted exponential decay curves (dotted white). First, the lattice plane spacing after relaxation ($d_{\text{RHEED}}^{\text{relax}}$) is calculated. The values, reported in Table 1, are very similar for the two samples (variation below 1%). Still, it decreases as a function of the Ge flux, which is once more consistent with the incorporation of GeTe in the alloys.

It is the beginning of the growth, however, which unveils a fundamental similarity between the samples. For both GST alloys, the lattice spacing at nucleation is slightly larger than that of bulk Sb_2_Te_3_ (dashed blue line in Figure 5e).^[^ ^41^ ^]^ This could be related to a small excess of Sb at the interface with Si, leading to the initial formation of Sb_2 +*x*_Te_3_.^[^ ^14^, ^53^ ^]^ A similar nucleation process was reported for epi‐GeTe on Sb‐passivated Si(111), while full relaxation occurred after the deposition of two GeTe BLs.^[^ ^54^ ^]^ Interestingly here, the relaxation of the lattice spacing depends upon the film composition. Indeed, the decay lengths of the exponential fits *L*_decay_ are very close to the average GST block thicknesses (see Table 1), suggesting the existence of a univocal mechanism of strain release in layered epi‐GST. For GST225 (yellow curve in Figure 5e) an almost full relaxation occurs within the first 5 nm, corresponding to ≈3 blocks, while for GST528 (dark red curve in Figure 5e) the final lattice spacing is reached after 10 nm, close to 4 blocks (see Ge_2_Sb_2_Te_5_ and Ge_5_Sb_2_Te_8_ blocks schematic in the figure). At this point, the mismatch between the material initially nucleated at the interface with Si and the growing GST film has to be emphasized. In fact, it increases as more GeTe is incorporated in the alloy. This consideration nicely addresses qualitatively the different number of average GST blocks required for GST225 and GST528 to reach their final lattice spacings.

The present results differ substantially from the case of pure covalently bonded materials, for which the relaxation thickness is inversely proportional to the mismatch with the substrate underneath.^[^ ^55^ ^]^ Accordingly, a deviation of the growth from a quasi‐vdW epitaxy regime toward a covalently bonded one, forced by the reducing number of ordered vacancy layers per unit length, does not occur. Therefore, a partial 2D character needs to be preserved. Notably, an unparalleled relaxation process was found in Sb_2_Te_3_/GeTe heterostructures,^[^ ^56^, ^57^ ^]^ led by a deviation of such structures from pure weakly interacting systems. Hence, the relaxation presented in this study points to a similar role of the epi‐GST layered structure in the determination of the inter‐block coupling and strain release.

## 5 Conclusion and Outlook

We reported on the structural and ferroelectric properties of epitaxial GST alloys with GeTe‐rich composition. The material, fabricated by MBE, was investigated by a combination of RHEED, XRD, STEM, and PFM techniques. We demonstrated the tuning of the average epi‐GST block size by changing the film composition from GST225 to GST528. The analysis of the layered structure of epi‐GST based on STEM images revealed an unexpected distribution of block size, stabilized by epitaxy, in the sample with high GeTe content. The accurate description of the complex stacking held by the samples fostered the development of dedicated XRD simulations. We discovered an intriguing dependence of the atomic distances of the (GeTe)_*m*_(Sb_2_Te_3_)_1_ blocks on their average thickness in the films with different composition.

The layered structure of epi‐GST, as detailed by our analysis, plays a primary role in the relaxation of the strain observed during growth. Indeed, a signature of the partial 2D character of the films is found, regardless of their average block size. That is, the reduced number of vdW gaps per unit length in GeTe‐rich GST is still sufficient to confer properties between covalently bonded and 2D materials. In other words, our data shed light on the resilience of quasi‐vdW epitaxy, even for GST lamellae as thick as 33‐atomic layers. This is an impressively large number as compared to other families of layered materials, which paves the way for the realization of novel vdW heterostructures and the further exploitation of strain engineering in weakly coupled systems. At the same time, it highlights the strength of the MBE technique in the design of Ge–Sb–Te materials with unique properties.

Most importantly, we sharpened our analysis of the morphology of (GeTe)_*m*_(Sb_2_Te_3_)_1_ lamellae at the atomic scale. We succeeded in accurately recording the atomic structure of GST lamellae from high resolution STEM micrographs. These data are confirmed by our modeling. Indeed, DFT calculations and XRD simulations jointly unearthed the unusual symmetry of GST slabs as a function of lamellar size. i) In thin lamellae the Peierls distortion at each GeTe BL is globally neutralized by the mirror symmetry at the center of the blocks. That is, an FE domain wall is locked in between the two halves with opposite polarizations but equal in size. ii) On the contrary, the inversion symmetry is broken in very thick GST lamellae, which yields a global net FE polarization to be a third of that measured in epi‐GeTe. A similar asymmetric geometry was reproduced by theory, which also suggested the formation of a tilted (GeTe)_*m*_ slab sandwiched in the lamella by SbTe capping layers on both sides, closely resembling the behavior of other 2D FE materials.

The analysis at the local scale was complemented by PFM and electroresistive measurements, corroborating the composition‐dependent ferroelectric behavior of epi‐GST. We found a reduction of the piezomechanical response from pure GeTe to GeSbTe alloys, up to the complete disappearance of the signal in sample GST225, in which thick lamellae are not present.

Ultimately, the thick GeTe‐rich lamellae uncovered in this work constitute an attractive point of junction between phase‐change and FE switching functionalities, which might be exploited in innovative devices. At the same time, this approach offers the possibility to tune the FE properties of GeTe in a 2D object, potentially facilitating the switching of spin‐to‐charge conversion in spintronic devices^[^ ^27^ ^]^ and its integration in vdW heterostructures. A challenge for future research is the tuning of the epi‐GST block distribution toward alloys even richer in GeTe, while tackling the retainment of a layered structure.

## 6 Experimental Section

### Sample Preparation

GST films were fabricated by MBE on Si(111)‐($\sqrt{3}\times \sqrt{3}$)R30°‐Sb passivated surfaces. The substrate cleaning method and the preparation of the Si(111)‐($\sqrt{3}\times \sqrt{3}$)R30°‐Sb surface was described by Wang et al.^[^ ^58^ ^]^ For sample GST225, flux ratios of ≈0.5 and ≈1.8 were used for Ge/Sb and Te/Sb, respectively.^[^ ^31^ ^]^ For the GeTe‐rich sample (GST528), the alloy composition was controlled by increasing the atomic flux of Ge (see Table 1). All other growth parameters were unchanged. For both experiments, the substrate temperature was ≈230 °C and the deposition time was 90 min. The RHEED movies were recorded in the first 60 min of deposition.

### Transmission Electron Microscopy

Cross‐sectional specimens were prepared with a Thermo Fisher Scientific Helios G4 CX, using gradually decreasing acceleration voltages of 30, 5, and 2 kV. In the final step the specimen was polished for 40–80 s at 0.2 kV on both sides with Ar+ using a Gatan PIPS II. TEM analyses were performed with a double aberration corrected FEI Themis Z, operated at 300 kV. HAADF‐STEM images were recorded with a probe current of 50 pA, convergence semi‐angle 21 mrad and HAADF collection angles 61–200 mrad. For better visibility, micrographs in panels (b) and (c) of Figure 5 were filtered with the average background subtraction filter, freely available at http://www.dmscripting.com/hrtem_filter.html. Details about GPA analysis are reported in the Supporting Information. For the analysis in Figure 1, the atomic positions were first fitted in orthogonally rotated defect‐free portions of the GST lamellae. Then, the atomic plane distance profiles were calculated for each atomic chain along the direction indicated by the black arrow and averaged. The errors in the numerical fitting of the atomic positions are shown in Figure 1 and Figure S1, Supporting Information.

### Density Functional Theory Calculations

Calculations were performed within density functional theory (DFT) as implemented in the Quantum‐Espresso suite of programs.^[^ ^59^ ^]^ The Perdew–Becke‐Ernzerhof exchange and correlation functional^[^ ^60^ ^]^ and the semiempirical correction due to Grimme (D2)^[^ ^61^ ^]^ were used to include van der Waals interactions. Norm conserving pseudopotentials and a plane wave expansion of Kohn–Sham orbitals up to an energy cutoff of 35 Ry were employed. Brillouin zone integration for electronic structure calculations was performed over uniform meshes of size from 3 × 3 × 3 to 6 × 6 × 1.

The disorder was introduced in the Ge/Sb sublattices as described in Figure 2. The Ge/Sb distribution for Ge_2_Sb_2_Te_5_ was taken from the experimental data in ref. [34] and it corresponded to 50% occupation of Sb in the outermost cationic layer (Matsunaga model). The Ge/Sb distribution for Ge_3_Sb_2_Te_6_ corresponded to that reported in ref. [35]. No experimental data were available on the Ge/Sb distribution in trigonal Ge_4_Sb_2_Te_7_, therefore a configuration that avoids the formation of pure Ge layers was chosen. In the model of Ge_5_Sb_2_Te_8_, the presence of a pure Ge layer on the second cationic layer closer to the vdW gap was chosen in analogy with the structure proposed experimentally for Ge_6_Sb_2_Te_9_.^[^ ^36^ ^]^ Instead, a Ge/Sb distribution close to that of Ge_3_Sb_2_Te_6_ led to a phonon instability at the Γ‐point. It was remarked that, also in Ge_3_Sb_2_Te_6_, disorder in the Ge/Sb sublattices was mandatory for the system to be dynamically stable. The crystal with only Sb in the outermost layers (Kooi structure) was dynamically unstable with negative frequency phonons at the Γ‐point, as shown in a previous DFT work.^[^ ^62^ ^]^ As shown in Figure 2e, in order to stabilize the Ge_6_Sb_2_Te_9_ model the disorder in the Ge/Sb sublattices had to be further increased with respect to the distribution proposed experimentally in ref. [36].

With these constraints on the Ge/Sb composition on each layer, the distribution of Ge/Sb atoms was chosen in such a way as to fulfill the rules proposed by Da Silva et al.^[^ ^40^ ^]^ as much as possible. According to ref. [40] the more stable structures were those in which a central Te site was surrounded by three Ge neighbors and three Sb neighbors which corresponded to the so‐called 3Ge‐Te‐3Sb rule. The second rule was that the more stable structures had Ge sites and Sb sites on opposite sides of the octahedral Te sites.

The supercells (hexagonal or trigonal) contained a number of atoms equal to four times the number of atoms in the formula unit and four atoms in each hexagonal layer. The average interplanar distances as shown in Table 2 and Table S2, Supporting Information, were obtained by assigning an average position along *z* (the *c* hexagonal axis) of atoms belonging to the same plane.

The structure of a Ge_10_Sb_2_Te_13_ thick lamella (*m* = 10) was also investigated by means of DFT calculations. The block was initially modeled as a centrosymmetric Kooi‐like structure, using a trigonal cell which contained 25 atomic layers with space group R$\bar{3}$m. We consider a Kooi‐like occupation of the cationic sublattice, that is, no occupational disorder, for computational reasons. In this case, in fact, the simulation cell contained only one atom per layer. The structure was hence fully relaxed to a stable configuration that breaks inversion symmetry. The equilibrium structure still displayed on average a hexagonal cell geometry with lattice parameters *a* = 4.22 Å and *c* = 132.38 Å. The calculations associated with this structure had been performed adopting the same computational scheme as above, using a 4 × 4 × 4 Monkhorst–Pack mesh along with a kinetic energy cutoff of 35 Ry. Variable‐cell simulations were carried out using a tighter cutoff of 65 Ry. Similar results were obtained for a lamella with *m* = 11, corresponding to 27 atomic layers. All the details are reported in the Supporting Information.

### Piezoresponse Force Microscopy

PFM measurements were performed using a Keysight 5600LS AFM employing conductive tips manufactured by Applied NanoStructures Inc. (AppNano ANSCM‐PT, n‐doped single crystal Si with a platinum coating; *L* = 225 μm, *k* = 3 N m^−1^). Imaging of FE domains was done at remanence (no DC voltage), exciting the conductive tip using an AC voltage with a typical frequency of about 60 kHz and amplitude in the range 1–2 V, not to exceed the coercive voltage of the material under test. The response of the ferroelectric material was determined by demodulating the AC deflection of the tip with a dual‐phase lock‐in amplifier able to record the *X* and *Y* output signals. After the application of a proper procedure that removed background contributions,^[^ ^63^ ^]^ *X* contained the complete piezomechanical response. From the PFM signal *X*, it was possible to estimate the piezoelectric coefficient *d*_33_ in the out‐of‐plane direction, which linked mechanical deformation and applied voltage, using the following formula, valid for a working frequency well‐below the contact resonance: $d_{33}=\frac{|X|\cdot S}{V_{\text{AC}}}$, where *S* is the tip sensitivity in nm V^−1^ whereas *V*_AC_ is the applied AC voltage. The tip sensitivity was 209 nm V^−1^, as measured from force–distance curves. In order to ensure the comparability of measurements performed among different samples and thus avoid any experimental bias due to modifications of the response of the tip, the correct operating conditions were checked on the pure GeTe reference sample each time.

### Electroresistive Measurements

Ferroelectric gating was performed at room temperature on epitaxial GeTe and GST samples. Contacts made of 100 nm thick Ti (40 × 40 μm^2^) were directly deposited by electron‐beam evaporation on the film surface using a proper shadow mask. Square voltage pulses with a duration of 10 ms were generated by a Keithley 2611B source‐and‐measure unit to switch the FE polarization. Afterward, to retrieve associated resistance univocally linked to ferroelectric polarization,^[^ ^27^ ^]^ the *I*–*V* curves at relatively low voltages (<1 V) were measured with the same instrument.

### X‐Ray Diffraction

The diffractometer used for the XRD and XRR characterization is a PANalytical X'Pert PRO Materials Research Diffractometer system with a Ge(220) hybrid monochromator, employing a CuK_α1_ (λ = 1.540598 Å) X‐ray radiation. The data analysis was carried out using *X‐rayutilities*.^[^ ^64^ ^]^ The estimation of the GST composition based on the measured average block size did not take into account residual disordered vacancies in the films. Therefore, a small deviation of the atomic composition could not be targeted.

### Reflection High‐Energy Electron Diffraction

The RHEED pattern of the initial Si(111)‐($\sqrt{3}\times \sqrt{3}$)R30°‐Sb surface, taken along a $\left\langle 1\bar{1}0\right\rangle$ azimuth, was used as a reference for the conversion to *Q*‐space. The $\left\{11\bar{2}\right\}$ lattice plane spacing *d*_RHEED_ of the growing GST films was calculated from the *Q*‐separation of the main streaks ($\frac{4\pi }{\Delta Q_{\text{main}}}$).^[^ ^52^, ^56^, ^57^, ^65^ ^]^ The thickness values on the *x*‐axis in Figure 5e were estimated by fitting X‐ray reflectivity (XRR) curves (not shown), assuming for simplicity a constant growth rate during each deposition. The experimental curves were fitted using an exponential decay model including an offset representing the relaxed lattice spacing.

## Conflict of Interest

The authors declare no conflict of interest.

## Author Contributions

S.C. designed the experiments with contributions from R.C. S.C. performed the MBE growths. S.C. carried out RHEED and XRD characterization, supported on the analysis by D.K. and F.A. XRD simulations were carried out by S.C. with the support of D.K. and V.H. The code was implemented by D.K. and S.C. in analogy with that developed by V.H. for previous studies. J.M. performed the STEM measurements with the support of B.J.K. The analysis of atomic plane distances profiles was developed by J.M. and S.C. The DFT calculations were carried out by D.D. and O.A.E.K. with the support of M.B. F.F. and C.R. performed PFM and electroresistive measurements. All the authors discussed the results. The paper was written by S.C. with the help and contributions from all co‐authors.

## Supporting information

Supporting Information

## Data Availability

The data that support the findings of this study are available from the corresponding author upon reasonable request.
