# Supplementary material for: Thick Does the Trick: Genesis of Ferroelectricity in 2D GeTe‐Rich (GeTe) m (Sb2Te3) n Lamellae
Source: Adv Sci (Weinh). 2023 Nov 21;11(1):2304785. doi: 10.1002/advs.202304785 (PMC10767439; doi:10.1002/advs.202304785)
Supplement: Supplementary file 1 — Supporting Information [file ADVS-11-2304785-s001.pdf]

ADVANCED SCIENCE
Open Access

Supporting Information

for *Adv. Sci.*, DOI 10.1002/advs.202304785

Thick Does the Trick: Genesis of Ferroelectricity in 2D GeTe-Rich $(GeTe)_m(Sb_2Te_3)_n$ Lamellae

Stefano Cecchi, Jamo Momand, Daniele Dragoni, Omar Abou El Kheir, Federico Fagiani, Dominik Kriegner, Christian Rinaldi, Fabrizio Arciprete, Vaclav Holý, Bart J. Kooi, Marco Bernasconi and Raffaella Calarco*

Supporting Information
Thick does the trick: genesis of ferroelectricity in two-dimensional GeTe-rich $(GeTe)_m(Sb_2Te_3)_n$ lamellae

Stefano Cecchi Jamo Momand Daniele Dragoni Omar Abou El Kheir Federico Fagiani Dominik Kriegner Christian Rinaldi Fabrizio Arciprete Vaclav Holý Bart J. Kooi Marco Bernasconi Raffaella Calarco*

1 Density Functional Theory Calculations

The theoretical equilibrium lattice parameters of the different compounds are compared with experimental data from Refs. [1, 2, 3, 4] in **Table S1**. The average interplanar distances obtained from density functional theory (DFT) calculations to be compared with XRD simulation parameters are reported in **Table S2**.

Table S1: Theoretical (DFT) structural parameters of the hexagonal phase of crystalline $(GeTe)_m(Sb_2Te_3)_1$ compounds, i.e. $Ge_2Sb_2Te_5$ (Kooi and Matsunaga), $Ge_3Sb_2Te_6$, $Ge_4Sb_2Te_7$, $Ge_5Sb_2Te_8$ and $Ge_6Sb_2Te_9$ compared with experimental data (in parenthesis) from Refs. [1, 2, 3, 4].

m (#)		a (Å)	c (Å)
2	(Kooi)	4.191 (4.2) [1]	17.062 (17.2) [1]
	(Matsunaga)	4.184 (4.224) [2]	17.202 (17.239) [2]
3		4.181 (4.198) [3]	61.590 (62.021) [3]
4		4.171	72.653
5		4.168	27.591
6		4.165 (4.176) [4]	93.350 (93.396) [4]

Table S2: Atomic plane distances (Å) as defined in Table 2 for the theoretical models of disordered $Ge_2Sb_2Te_5$ (Matsunaga), $Ge_3Sb_2Te_6$, $Ge_4Sb_2Te_7$, $Ge_5Sb_2Te_8$ and $Ge_6Sb_2Te_9$. In particular, the d_2 parameter is the average of all inner Te-Te interplanar distance excluding the Te-Te distance closer to the vdW gap which is given by d_1. The d_{z1} distance is the interplanar distance between the outermost Te layer and the nearest Ge/Sb layer. The d_{z2} parameter is the average interplanar distance between the inner Te layers and the nearest inner Ge/Sb layer excluding the two Te-Ge/Sb interplanar distances at the center of the slab. Each interplanar distance is obtained by averaging over all atoms in the same layer.

m (#)		d_{vdW} (Å)	d_1 (Å)	d_2 (Å)	d_{z1} (Å)	d_{z2} (Å)
2	(Matsunaga)	2.93	3.61	3.52	1.57	1.69
3		2.94	3.67	3.40	1.65	1.67
4		3.02	3.64	3.47	1.56	1.62
5		2.96	3.65	3.48	1.57	1.56
6		3.00	3.66	3.46	1.56	1.59

The structure of a $Ge_{11}Sb_2Te_{14}$ block is also investigated by means of DFT calculations. The block is initially modelled as a thick centrosymmetric Kooi-like structure, using a hexagonal cell which contains 27 atomic layers

stacked with an ABC sequence along the c-axis. As for the lamella with $m = 10$, we consider a Kooi-like occupation of the cationic sublattice, i.e. no occupational disorder, for computational reasons. This structure shows very similar features as the computed $Ge_{10}Sb_2Te_{13}$ block. Indeed, it is also mechanically unstable and spontaneously relaxes to a new configuration which breaks inversion symmetry. The equilibrium structure, beyond numerical uncertainty, still displays an hexagonal cell geometry with lattice parameters $a = b = 4.20$ Å and $c = 47.44$ Å. The energy of this structure is 3 meV/at lower than the centrosymmetric starting configuration, and is mechanically stable as observed from the phonon frequencies calculated at the Γ point by density functional perturbation theory. [5] Moreover, the formation of a tilted GeTe BLs structure is again found. In this case, the tilt angle of the GeTe BLs is ≈69° with respect to the direction normal to the lamella. We also verified that such a configuration is 5 meV/at lower in energy relative to a fully relaxed Ferro-like configuration, which corresponds to a $(GeTe)_{11}$-Sb_2Te_3 heterostructure. The calculations associated with these structures have been performed adopting the same computational scheme as that described in the main text, using a 8×8×2 Monkhorst-Pack mesh along with a kinetic energy cutoff of 28 Ry. Variable-cell simulations are carried out using a tighter cutoff of 50 Ry.

In order to confirm the formation of a tilted bulk-like $(GeTe)_m$ slab in the thick GST lamellae, we computed for comparison a bulk GeTe crystal by DFT. The calculated cell parameters are $a_{trig} = 4.208$ Å and $\alpha = 58.99°$. In **Table S3** we compare the bilayer (BL) plane thicknesses and Ge-Te bond lenghts of the three structures.

Table S3: Comparison between the structure of the simulated tilted $(GeTe)_{10}$ and $(GeTe)_{11}$ slabs encapsulated in the thick GST lamellae and the bulk GeTe. In particular, $d^{BL}_{Te\text{-}Te}$ is the distance between Te planes defining the BL structure, $d^{BL}_{Ge\text{-}Te}$ is the short distance between Ge and Te planes in the BL, $l^{short}_{Ge\text{-}Te}$ and $l^{long}_{Ge\text{-}Te}$ are the lengths of short(long) Ge-Te bonds.

	$d^{BL}_{Te\text{-}Te}$ (Å)	$d^{BL}_{Ge\text{-}Te}$ (Å)	$l^{short}_{Ge\text{-}Te}$ (Å)	$l^{long}_{Ge\text{-}Te}$ (Å)
$(GeTe)^{lamella}_{10}$	3.47 ± 0.01	1.48 ± 0.05	2.82 ± 0.02	3.12 ± 0.03
$(GeTe)^{lamella}_{11}$	3.440 ± 0.002	1.54 ± 0.02	2.84 ± 0.02	3.07 ± 0.03
$GeTe_{bulk}$	3.462	1.501	2.825	3.093

2 Symmetry Breaking and Ferroelectric Polarization

The local ferroelectric (FE) distortion along the $(GeTe)_m(Sb_2Te_3)_1$ slabs, as shown in Figure 1 and Figure 2, is calculated using the Equation:

$$FE^{(j)} = \frac{d^{(j)}_{Ge/Sb} - d^{(j)}_{Te}/2}{d^{(j)}_{Te}} \tag{1}$$

where $d^{(j)}_{Ge/Sb}$ is the distance between the jth Te and Ge/Sb planes and $d^{(j)}_{Te}$ is the distance between the jth and $(j+1)^{th}$ Te planes, with j integer between 0 and $m+1$. The resulting total FE distortion is calculated as follows:

$$FE_{tot} = \sum_{j=0}^{m+1} FE^{(j)} \tag{2}$$

In **Table S4** we report the values of FE_{tot} calculated for the measured lamellae, along with the average FE distortion per BL equivalently measured in an epitaxial GeTe film. To better compare the data, the average FE distortion per BL ($\frac{FE_{tot}}{m}$) calculated for the measured lamellae is shown in parenthesis.

Table S4: Total FE distortion calculated for the measured lamellae. The average FE distortion per BL is shown in parenthesis. The average BL FE distortion measured in an epitaxial GeTe film is included for comparison.

m (#)	FE_{tot} (1)
3	-0.0080 (-0.0027)
4	-0.0083 (0.0021)
5	0.0195 (0.0039)
6	-0.0095 (-0.0016)
10	0.1715 (0.0172)
13	0.3813 (0.0293)
GeTe BL	-0.0532

The atomic plane distances measured in a thick GST lamella with $m = 13$ is shown in **Figure S1**. The asymmetry exhibited in the block is qualitatively comparable with that in Figure 1(e) in the main text. Interestingly, the local FE distortion at the lamella bottom edge is the largest we have measured so far, while its overall distortion FE_{tot} is slightly above half of that of an equivalent number of GeTe BLs measured in an epitaxial sample.

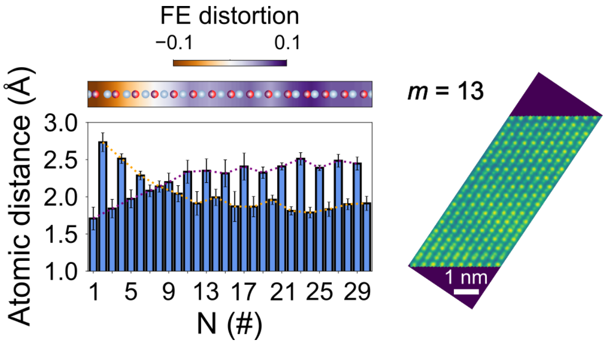

Figure S1: Atomic plane distances (light blue bars) of a GST block with $m = 13$, extracted from high resolution STEM micrographs of sample GST528. Te and Ge/Sb atoms are sketched in gray and violet/red, respectively. The portion of lamella used for the analysis is also shown. The colormap represents the FE distortion along the slab. Dotted orange and purple lines highlight the presence of a FE domain wall, which is displaced from the center as for the block with $m = 10$ in Figure 1(e).

3 Local Ferroelectric response of $(GeTe)_m(Sb_2Te_3)_n$

The FE behavior of GST samples was also tested locally, measuring ferroelectric hysteresis loops by vertical piezoresponse force microscopy (PFM). The out-of-plane component of the polarization was measured at a low frequency (far below the contact resonance of the tip). The poling DC bias and the probing AC voltage were applied to the tip in pulsed DC mode, as done for epitaxial GeTe in Ref. [6]. As exemplarily shown in **Figure S2** for sample GST528, the PFM amplitude and phase show a clear ferroelectric response, with the minimum of the amplitude in correspondence with the FE coercive field, and a reversal of the phase for inward and outward polarization. However, the effective amplitude of each loop collected locally at the surface could present some variability. Thus, the quantification of the overall response for each composition in the main text is calculated as a mean value over a relatively large surface by domain patterning and subsequent imaging.

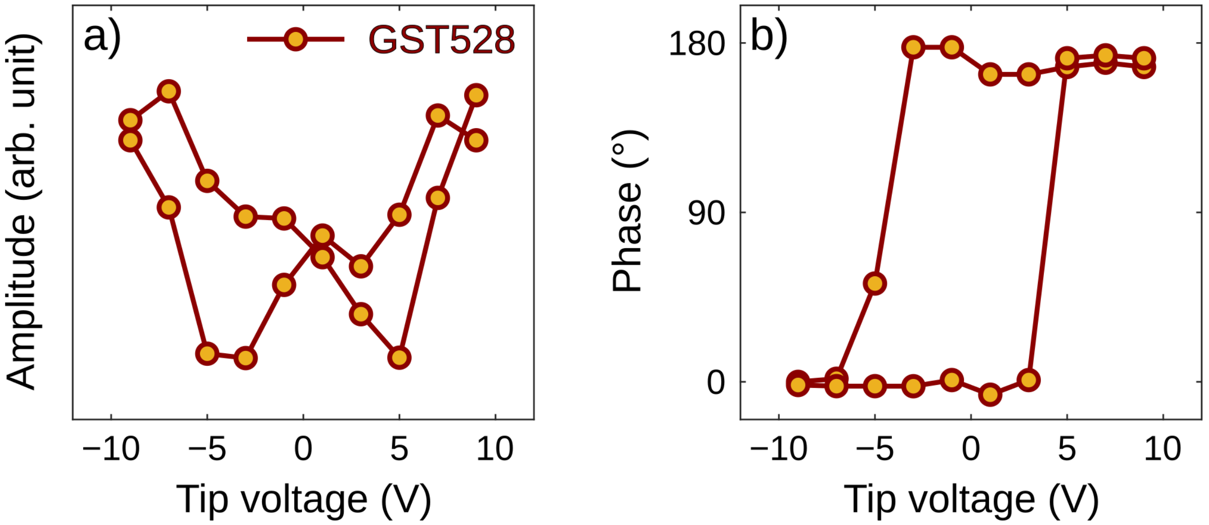

Figure S2: FE hysteresis measured by piezoresponse force microscopy on sample GST528. (a) Amplitude and (b) phase collected on the film surface at a frequency of 60 kHz. The out-of-plane FE polarization is evident from the 180° change in the phase and from the behavior of the amplitude.

4 Scanning Transmission Electron Microscopy

The method applied to process HAADF-STEM micrographs and extrapolate the block atomic-plane distribution in the GST films is similar to that presented in a previous publication by Momand et al.. [7] The steps of the analysis are shown in **Figure S3**(a-d) for a large scale image of sample GST225. The resulting block distribution is equivalent to those used in the main text.

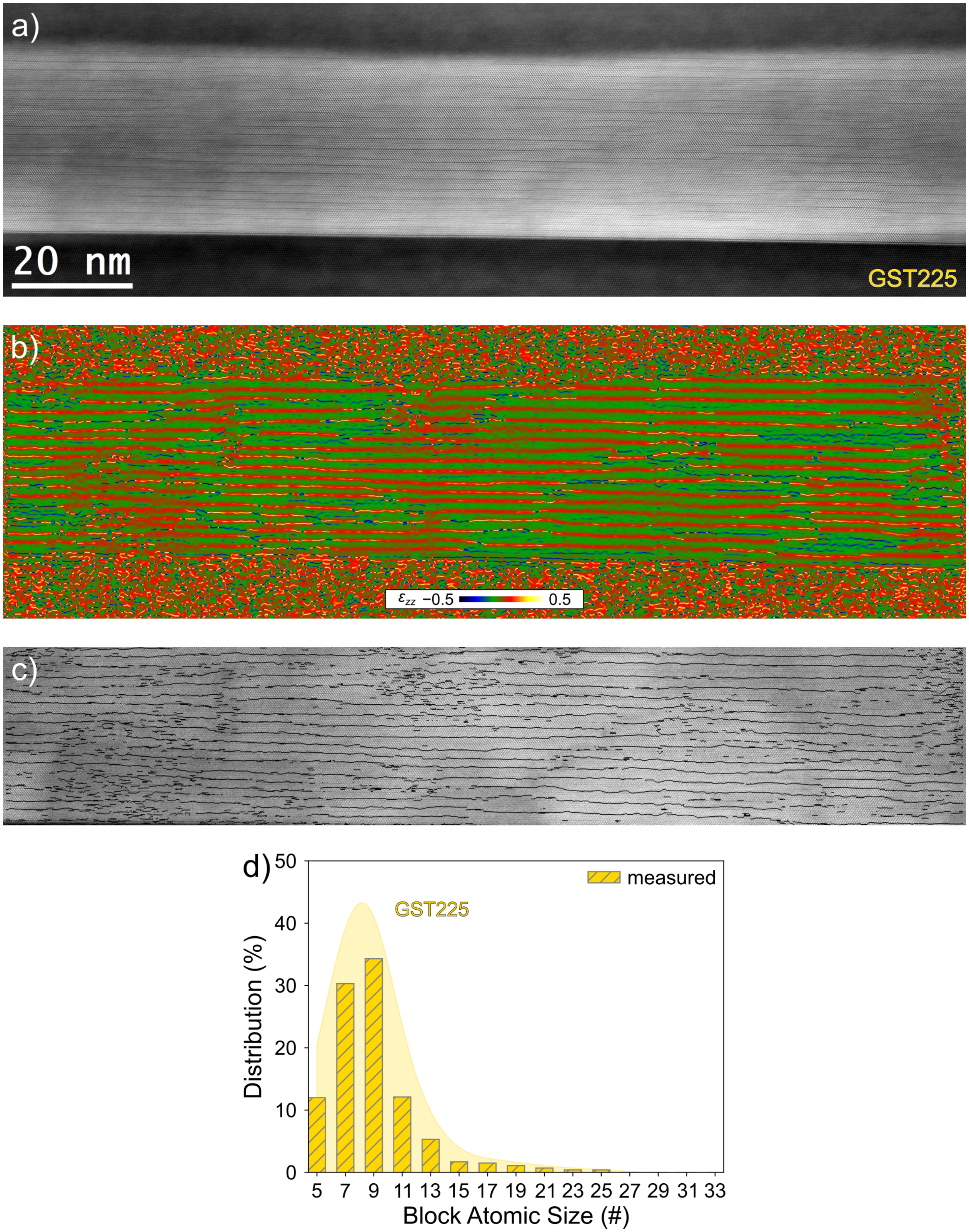

Figure S3: (a) HAADF-STEM micrograph and (b) its GPA ϵ_{zz} map of a large scale image of GST225 sample. (c) Mapping of the vdW gaps as obtained after processing. (d) Resulting distribution of $(GeTe)_m(Sb_2Te_3)_1$ block atomic planes. The kernel density estimate of the distribution is plotted in the background as guide for the eye.

5 X-Ray Diffraction Simulations

Our simulations of the diffraction signal of the $(GeTe)_x(Sb_2Te_3)_1$ thin films are based on the kinematical theory description of random chains of $(GeTe)_m(Sb_2Te_3)_1$ building blocks of various length. As indicated in **Figure S4** for GST blocks with an odd(even) number of Te planes, a building block is defined by a Te-Sb-Te-$(Ge\text{-}Te)_m$-Sb-Te set of lattice planes. The integer variable m determines the length of the inserted $(Ge\text{-}Te)_m$ slab and at the same time corresponds to the chemical composition $(GeTe)_m(Sb_2Te_3)_1$ of the lamella. The building blocks are separated by a vdW gap (d_{vdW}). Combining building blocks with different m with appropriate probabilities any sample compositions $(GeTe)_x(Sb_2Te_3)_1$ can be modelled. Inside a building block, the atomic spacings are described by the separation of Te layers adjacent to a vdW gap (d_1) and those in the center of the block (d_2). The distances of the Sb and Ge layers from the nearest Te layer below are described by d_{z1} and d_{z2}, respectively. The present model with five atomic spacings includes all free paramenters for blocks up to five Te layers. Clearly, it is a simplification for larger blocks, which is, however, required since the amount of features originating from the GST films is limited in the experimental data. As shown in Figure S4(b), in GST blocks with an even number of Te planes a Ge plane occupies the center and is separated by $d_2/2$ from the nearest Te planes. The diffraction intensity is calculated in complete analogy to equations 15 and 16 in the work of Steiner et al.. [8]. Using the five atomic plane distances described in this paragraph, two Debye Waller factors for Te and Ge/Sb planes accounting for the disorder in Ge/Sb sublattices, as well as scaling parameters for primary beam intensity and background, we fitted the model curves to our experimental data. The distribution of block length given by the variable m was determined from the STEM data and used as fixed input parameter in our model.

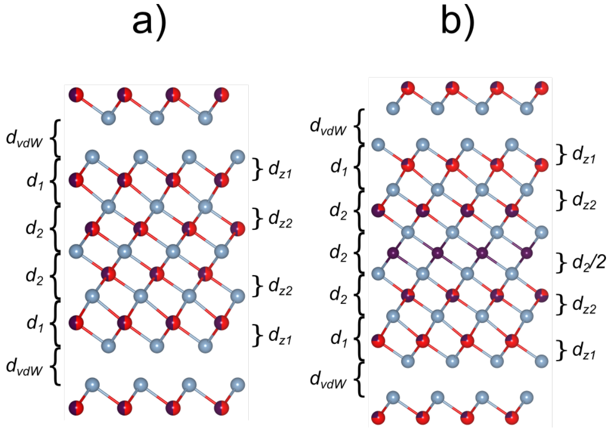

Figure S4: Schematic of the five atomic distances mirror-symmetric structure of $(GeTe)_m(Sb_2Te_3)_1$ building blocks employed in the XRD simulations. The sketches are valid for GST lamellae with (a) an odd number of Te layers (m even) and (b) an even number of Te layers (m odd).

References

[1] B. J. Kooi, J. T. M. De Hosson, *Journal of Applied Physics* **2002**, *92*, 7 3584.

[2] T. Matsunaga, N. Yamada, Y. Kubota, *Acta Crystallographica Section B Structural Science* **2004**, *60*, 6 685, tex.ids: matsunaga_2004d tex.isbn: 0108768104 tex.publisher: International Union of Crystallography.

[3] T. Matsunaga, R. Kojima, N. Yamada, K. Kifune, Y. Kubota, M. Takata, *Applied Physics Letters* **2007**, *90*, 16 161919.

[4] T. Matsunaga, H. Morita, R. Kojima, N. Yamada, K. Kifune, Y. Kubota, Y. Tabata, J.-J. Kim, M. Kobata, E. Ikenaga, K. Kobayashi, *Journal of Applied Physics* **2008**, *103*, 9 093511.

[5] S. Baroni, S. de Gironcoli, A. Dal Corso, P. Giannozzi, *Reviews of Modern Physics* **2001**, *73*, 2 515, arXiv: 0012092v1 [arXiv:cond-mat] tex.arxivid: arXiv:cond-mat/0012092v1 tex.isbn: 0034-6861.

[6] C. Rinaldi, S. Varotto, M. Asa, J. Sławińska, J. Fujii, G. Vinai, S. Cecchi, D. Di Sante, R. Calarco, I. Vobornik, G. Panaccione, S. Picozzi, R. Bertacco, *Nano Letters* **2018**, *18*, 5 2751.

[7] J. Momand, R. Wang, J. E. Boschker, M. A. Verheijen, R. Calarco, B. J. Kooi, *Nanoscale* **2017**, *9*, 25 8774.

[8] H. Steiner, V. Volobuev, O. Caha, G. Bauer, G. Springholz, V. Holý, *Journal of Applied Crystallography* **2014**, *47*, 6 1889.
